# Preparation of Antioxidant and Antibacterial Chitosan Film from *Periplaneta americana*

**DOI:** 10.3390/insects12010053

**Published:** 2021-01-11

**Authors:** Sicong Chen, Xunfan Wei, Zhuoxiao Sui, Mengyuan Guo, Jin Geng, Jinhua Xiao, Dawei Huang

**Affiliations:** Institute of Entomology, College of Life Sciences, Nankai University, Tianjin 300071, China; 1120200481@mail.nankai.edu.cn (S.C.); 1120180391@mail.nankai.edu.cn (X.W.); 1120190479@mail.nankai.edu.cn (Z.S.); 2120190992@mail.nankai.edu.cn (M.G.); 2120190985@mail.nankai.edu.cn (J.G.)

**Keywords:** *Periplaneta americana*, chitosan film, antioxidant, antibacterial activity

## Abstract

**Simple Summary:**

The American cockroach (*Periplaneta americana*) is a kind of insect distributed worldwide. Commonly, it is considered as a pest. However, nowadays, it has been developed as a potential resource of protein, lipid, and antibacterial peptide. Besides, it also contains chitin, which could be used to produce chitosan by deacetylation. Chitosan is a valuable biomaterial containing amino groups, and has been applied in various fields. However, the researches focusing on the applications of *P. americana* chitosan are rare, which might hinder the exploration of the value of *P. americana.* In this paper, we prepared and characterized the chitosan film from *P. americana*. The performances relating to food packaging of the obtained film were also examined. As the results showed, *P. americana* chitosan film could resist UV light effectively. It could also keep scavenging 2,2-diphenyl-1-picrylhydrazyl (DPPH) radicals in 8 h, proving its ability of antioxidant. In addition, it exhibited antibacterial activity by resisting the growth of *Serratia marcescens* and *Escherichia coli*. The results showed that *P. americana* chitosan film could work as a potential food packaging material, which implicated the value of *P. americana* chitosan and provided a new clue for the exploration of the value of more insects, especially pests.

**Abstract:**

Among different insects, the American cockroach (*Periplaneta americana*) has been bred in industrial scale successfully as a potential resource of protein, lipid, and antibacterial peptide. However, the application of its chitosan has not been studied widely, which has hindered the sufficient utilization of *P. americana*. In this paper, the chitosan from *P. americana* was separated, characterized, and processed into film (PaCSF) to examine its potential of being applied in food packaging. As the results of different characterizations showed, PaCSF was similar to shrimp chitosan film (SCSF). However, concerning the performances relating to food packaging, the two chitosan films were different. PaCSF contained more water (42.82%) than SCSF did, resulting in its larger thickness (0.08 mm). PaCSF could resist UV light more effectively than SCSF did. Concerning antioxidant activity, the DPPH radical scavenging ability of PaCSF increased linearly with time passing, reaching 72.46% after 8 h, which was better than that of SCSF. The antibacterial activity assay exhibited that PaCSF resisted the growth of *Serratia marcescens* and *Escherichia coli* more effectively than SCSF did. The results implied that *P. americana* chitosan could be a potential raw material for food packaging, providing a new way to develop *P. americana*.

## 1. Introduction

Since the global population is soaring, people are exploring more renewable resources to meet the demands of modern life. Recently, insects have attracted researchers’ focuses because they possess extraordinarily huge biomass, and they are promising resources of protein, lipid, and chitin [1]. In addition, some species of insects provide special substances, like antimicrobial peptides, which make them more valuable [2]. The American cockroach (*Periplaneta americana*) is one of the world’s most thriving insects distributing in tropic, subtropic, and temperate regions. In China, *P. americana* has been used to deal with ulcers, wounds, edema hypochondriac pain, and palpitations for thousands of years [3]. Previous researches have proven that *P. americana* possesses several outstanding biological properties, including antibacterial ability, antioxidant, inflammatory, and anticancer [4,5,6]. Attracted to these characteristics, people in China have reared *P. americana* in large scale for researches and commercial applications. For now, various substances—including protein, fatty acid, antimicrobial peptide, chitin, and chitosan—have been separated from *P. americana* so that they could be studied and applied separately [7,8,9,10].

Chitin is the second most abundant natural polysaccharide after cellulose. Chitin linearly consists of *N*-acetyl-d-glucosamine and d-glucosamine units that are linked by β(1,4) glycosidic bonds [11]. Chitin exists mainly as a structural component in arthropods [12], some mollusks, and cell walls of fungi and algae [13,14,15]. Chitosan is the deacetylated derivative of chitin. During the deacetylation, *N*-acetyl-d-glucosamine units would be changed to d-glucosamine units. Because of the large number of amino groups distributing along the polysaccharide chains, chitosan possesses several excellent properties, such as biocompatibility, non-toxicity, biodegradability, and the ability of anti-cancer [16,17]. Therefore, chitosan has been applied in various fields: agriculture, waste water treatment, food industry, cosmetic, and biopharmaceutics [18].

In the food industry, packaging is the final step for food preservation. The effect of packaging depends largely on the performance of the packaging film [19]. Recently, several natural biomaterials have been developed to produce active food packaging film, including protein, polysaccharide, and lipid [20]. Among the polysaccharide, chitosan is utilized most popularly because the obtained film would inherit the outstanding properties of chitosan. These properties—mainly antioxidant and antibacterial activity—would enhance the performance of the obtained film in food packaging. Commercial chitosan generally comes from crab and shrimp shells [21]. However, producing chitosan from *P. americana* is also reasonable, because the properties of chitosan from various resources are different [22]. This diversity would result in various chitosan films that could be applied in many specific areas. For example, molecular weight and degree of deacetylation of chitosan influenced the mechanical properties and bioactivity of the obtained film seriously [23,24]. In addition, since some inland areas lack fishery resources, *P. americana* would supplement the raw material of chitosan. Moreover, the development of *P. americana* chitosan could add value to this “pest”, helping change people’s attitude to it.

To the author’s best knowledge, for now, the researches focusing on *P. americana* chitosan film were rare. Therefore, in this paper, chitosan was prepared from *P. americana* and then processed into film. For comparison, commercial shrimp chitin was also utilized to produce chitosan film by the same method. All the chitin, chitosan, and chitosan films were characterized by Fourier transform infrared spectroscopy (FTIR), X-ray Diffraction (XRD), and Scanning electron microscopy (SEM). The performances relating to food packaging of both chitosan films were examined. This paper aimed to develop the application of *P. americana* chitosan in food packaging and to assist the exploration of *P. americana* resource in a new way.

## 2. Materials and Methods 

### 2.1. Materials and Reagents

We used 10 kilograms of dead and dried adult *P. americana* that were donated by Good-doctor Pharmaceutical Group (Sichuan, China). HCl and H_2_O_2_ solution were purchased from Bohua Chemical Reagent Co., Ltd. (Tianjin, China). NaOH powder, glacial acetic acid, hexane, LiCl, DPPH, and Dimethylacetamide (DMAc) were bought from Meryer Chemical Reagent Co., Ltd. (Shanghai, China). The mixture of methyl orange and aniline blue was bought from Yuanye Bio-Technology Co., Ltd. (Shanghai, China). All the chemical reagents were analytical grade. Commercial shrimp chitin (reagent grade) was purchased from Sigma–Aldrich (Shanghai, China). Cling wrap (MVDPC50, made of polyethylene) was bought from Top Group (Wuxi, China).

### 2.2. Chitin Isolation

#### 2.2.1. Defatting

*P. americana* was pulverized by a grinder (YS-04B, Zhengde Machinery Co., Ltd., Beijing, China) for 1 min. Then, the powder was defatted using hexane as the solvent by Soxhlet extraction method in water bath at 80 °C for 3 h. The residual powder was dried in an air-drying oven at 65 °C for 8 h.

#### 2.2.2. Deproteinization

The defatted powder was deproteinized by 4 rounds of hot alkali bath. Briefly, in the first round, the powder (1 g) was blended with 30 mL of 0.5 M NaOH solution. The mixture was heated with stirring at 95 °C and 500 rpm for 20 min. The residual powder was separated by centrifuging at 4000 rpm for 20 s (Centrifuge 5804, Eppendorf, Hamburg, Germany), and was then rinsed by distilled water until neutral. In the second round, the residual powder after the first round was again blended with NaOH solution at the same temperature and stirring speed; while the volume, concentration of NaOH, and the reaction time were changed to 5 mL, 4 M, and 80 min, respectively. Then the residual powder was separated and rinsed as described above. The conditions of the third and the fourth round were the same as those of the second round, except that the reaction time were changed to 150 min and 30 min, respectively. After deproteinization, the residual powder was dried at 105 °C in the oven for 10 h.

#### 2.2.3. Demineralization

The deproteinized powder was demineralized by 5 mL of 0.5 M HCl at 60 °C and 500 rpm for 1 h. The residual powder was separated, rinsed, and dried, as described in Section 2.2.2. The obtained residual powder was *P. americana* chitin (PaC).

### 2.3. Chitosan Preparation

PaC was bleached by 5 mL of 10% H_2_O_2_ at 80 °C and 500 rpm for 3 h, followed by separating, rinsing, and drying at 80 °C for 24 h. Then, PaC was deacetylated by 5 mL of 25 M NaOH solution at 130 °C and 500 rpm for 2 h. The residual powder was separated, rinsed, and dried at 80 °C for 24 h. This process was repeated three times. The final residual powder was *P. americana* chitosan (PaCS). The commercial shrimp chitin (SC) was also deacetylated under the same conditions, then the shrimp chitosan (SCS) was obtained.

### 2.4. Chitosan Film Formation

PaCS film (PaCSF) and SCS film (SCSF) were produced by previous module-casting method [25]. Briefly, chitosan (1 g) was totally dissolved in 50 mL of 1% acetic acid solution at room temperature, followed by adding 1 g of glycerol. The solution was stirred until homogeneous, and was then degassed by an ultrasonic cleaner (AS3120A AUTO SCIENCE, Tianjin, China) for 1 h. After that, 25 mL of the solution was poured into a petri dish (diameter = 9 cm), and was then dried at 65 °C for 15 h. The obtained film was peeled carefully and preserved in a desiccator at room temperature for at least 24 h before being characterized and tested.

### 2.5. Characterization

#### 2.5.1. Viscosity-Average Molecular Weight (Mv)

Mv of the chitin was determined according to Hong et al. [26]. The chitin sample (0.03 g) was dissolved in 1 dL of 5% LiCl/DMAc (w/v). The intrinsic viscosity of the solution was measured by an Ubbelohde capillary viscometer (Thermocap two, Fungilab, Beijing, China) at 25 °C. The Mv was calculated by Mark–Houwink–Sakurada equation:

[η] = KMv^α^ (1)

where [η] was the intrinsic viscosity of the chitin sample; K = 7.6 × 10^−5^ dL/g and α = 0.95.

The Mv of the chitosan was determined according to Rinaudo et al. [27]. The chitosan sample (0.03 g) was dissolved in 1 dL of 0.2 M sodium acetate/0.3 M acetic acid solution. The intrinsic viscosity of the solution was calculated by Equation (1), with K = 7.6 × 10^−4^ dL/g and α = 0.76.

#### 2.5.2. FTIR

FTIR spectra were analyzed by a Thermo Fisher Scientific Nicolet iS50 spectrometer (Thermo Fisher Scientific, Waltham, MA, USA) with the frequency of 4000–400 cm^−1^ at a resolution of 4 cm^−1^. Degree of acetylation (DA) of the chitin was calculated by the following equation [28]:

DA (%) = (A_1320_/A_1420_ − 0.3822)/3.133 × 100% (2)

where A_1320_ and A_1420_ were the area of the peak at 1320 and 1420 cm^−1^, respectively.

The degree of deacetylation (DDA) of the chitosan was calculated by referring to previous report [29]. The chitosan sample (0.03 g) was dissolved thoroughly in 3 mL of 0.1 M HCl solution. Then 20 µL of the mixture of methyl orange and aniline blue was added. NaOH solution (0.1 M) was utilized to titrate until the color of the solution changed from purple to blue-green. DDA was calculated by the following equations:

NH_2_ = [(C_1_V_1_ − C_2_V_2_) × 16]/m × 100% (3)

DDA (%) = NH_2_/9.94 × 100% (4)

where NH_2_ (%) was the amino content of chitosan; C_1_ and C_2_ were the concentration of NaOH and HCl solution (M), respectively; V_1_ was the volume of HCl solution (L) used to dissolve chitosan; V_2_ was the consumed volume of NaOH solution (L) after titration; m was the mass of the chitosan sample (g); 16 was the mass of amino group (g) corresponding to 1 mole of HCl molecules; 9.94 was the theoretical amino content (%) of fully deacetylated chitosan.

#### 2.5.3. XRD

XRD was conducted by Rigaku SmartLab diffractometer at 30 mA, 40 kV. The scanning angle was from 5° to 45°. Crystallinity index (CrI) of the chitin and chitosan samples were calculated according to the following equation [30]:

CrI_110_ = [(I_110_ − I_am_)] × 100% (5)

where I_110_ was the maximum intensity at 2θ ≈ 19.3°; I_am_ was the intensity of amorphous diffraction at 2θ ≈ 13°.

#### 2.5.4. SEM

The surface morphology of the chitin, chitosan, and chitosan films were observed by a scanning electron microscopy (QUANTA 200, FEI, Hillsboro, OR, USA).

### 2.6. Performances of Chitosan Films

#### 2.6.1. Film Thickness, Light Transmittance, and Opacity

Film thickness was measured by a digital caliper (precision = 0.01 mm).

Light transmittance of the chitosan films were determined according to Yang et al. [31]. SpectraMax Gemini EM was applied to scan the chitosan films from 200 to 800 nm. One piece of cling wrap (CW) was scanned under the same condition for comparison. The scanning speed was 10 nm/s.

The opacity of the chitosan films were calculated by the following equation [32]:

Opacity = A_600_/d (6)

where A_600_ was the absorbance of the film at 600 nm; d was the film thickness (mm).

#### 2.6.2. Water Content and Water Vapor Permeability (WVP)

The chitosan films were dried in the oven at 105 °C for 12 h. The water content was calculated by the following equation:

Water content (%) = (M_0_ − M_d_)/M_0_ × 100% (7)

where M_0_ and M_d_ were the weight of the film (g) before and after drying, respectively.

WVP was determined as described in previous report with a little modification [33]. The chitosan films and CW were utilized to seal a centrifuge tube of 15 mL containing 12 g of calcium chloride anhydrous. The tube was placed in a desiccator containing distill water at 20 °C. The tube was weighed at the interval of 24 h for 6 days. The WVP was calculated by the following equation:

WVP = (W × x)/(t × x)/(t × A × ∆P)(8)

where W was the increased weight of the tube (g); x was the film thickness (m); t was the time (s) consumed for that increase in the weight; A was the permeation area of the film (m^2^); ∆P was 2339 Pa at 20 °C.

#### 2.6.3. Antioxidant Activity Assay

DPPH radical scavenging assay was conducted to assess the antioxidant activity of the chitosan films and CW [33]. The chitosan films and CW were cut up, and 100 mg of each sample was immersed in 4 mL of 0.1 mM DPPH methanol solution. Another group of the same DPPH solution was set as Blank without any chitosan film or CW in it. After violently vibrating, the solution was kept in dark and the absorbance of the solution at 517 nm was determined at the interval of 1 h for 8 h. DPPH radical scavenging activity was calculated by the following equation:

DPPH radical scavenging activity = (A_0_ − A_t_)/A_0_ × 100% (9)

where A_0_ and A_t_ were the absorbance of Blank and the reacting solution, respectively.

#### 2.6.4. Antibacterial Activity Assay

*Serratia marcescens* and *Escherichia coli* were utilized to assess the antibacterial activity of the chitosan films by paper diffusion method [20]. The bacteria were activated in liquid lysogeny broth (LB) medium at 37 °C, and were then transferred and spread uniformly on the surface of LB agar plate. After that, one piece of disc paper (diameter = 6 mm) was set on the plate, and 20 µL of the film formation solution was dropped on the disc paper. For comparison, the solution without chitosan dissolved in was set as Blank (negative control); while 20 µL of 10 µg/µL gentamicin solution was set as positive control [34]. The agar plate was incubated at 37 °C for 12 h, then the inhibition zones were measured. 

### 2.7. Statistical Analysis

The experimental data were analyzed by SPSS 20 software (IBM, Armonk, NY, USA). The data were expressed as the average ± standard deviation of three replications. One-way analysis of variance (ANOVA) was utilized to measure the significant differences by Duncan’s multiple range at *p* < 0.05. 

## 3. Results and Discussion

### 3.1. The Changes of Characteristics from Chitin to Chitosan Film

#### 3.1.1. FTIR

**Figure 1:**
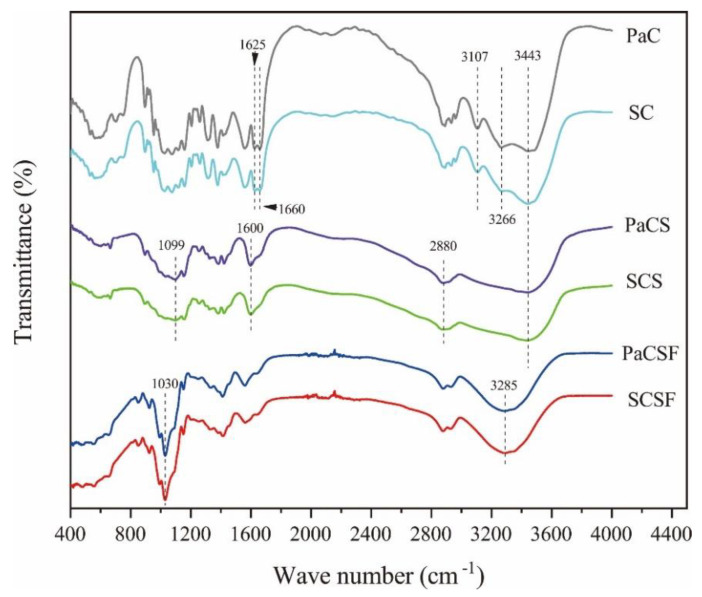
FTIR spectra of PaC, PaCS, PaCSF, SC, SCS, and SCSF.

FTIR spectra of chitin, chitosan, and chitosan films are shown in Figure 1. PaC and SC exhibited similar peaks at 3443 and 3266 cm^−1^, which were ascribed to O-H stretching and N–H stretching, respectively. The peak at 3107 cm^−1^ was the characteristic of secondary amides of α-chitin [35]. We could also see splitting band between 1660 and 1625 cm^−1^, which derived from the hydrogen bonds between C=O and O6–H [36]. All these characteristics proved the evidences of α-chitin.

The splitting band mentioned above vanished in PaCS and SCS, because most of the acetylamino groups that contain C=O were eliminated during deacetylation. We could observe obvious peaks at 1600 cm^−1^ in both PaCS and SCS, which were the characteristics of amino groups [37], proving the success of deacetylation. This explanation was backed up by the high DDA of chitosan, as presented in Table 1. The peaks at 3443, 2880 (C–H stretching), and 1099 cm^−1^ (C–O stretching) were also the characteristics of chitosan [38].

In PaCSF and SCSF, the peaks at 3443 cm^−1^ shifted to 3285 cm^−1^ (N–H stretching) because of the formation of lots of N-H hydrogen bonds among amino groups, glycerol, and water [39]. Additionally, sharp peaks at 1030 cm^−1^ (C–O stretching) appeared in PaCSF and SCSF because of the addition of glycerol. FTIR spectra showed that chitin, chitosan, and chitosan film from *P. americana* were similar to their counterparts from shrimp shell.

#### 3.1.2. XRD

**Figure 2:**
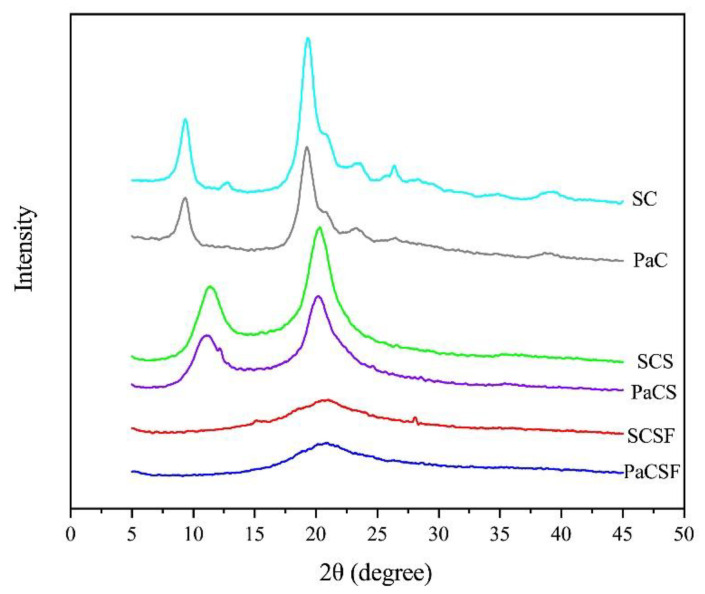
XRD patterns of PaC, PaCS, PaCSF, SC, SCS, and SCSF.

XRD was utilized to study the changes of structures, as presented in Figure 2. We could see typical crystalline peaks of α-chitin at 9.3° and 19.3° in both PaC and SC [26]. The two peaks became broader and weaker in PaCS and SCS, because the harsh conditions of deacetylation broke the structures of chitin. This result was supported by the values of CrI, which dropped significantly from chitin to chitosan (*p* < 0.05), as shown in Table 1.

PaCSF and SCSF presented much broader and weaker peaks at 21°, which were ascribed to the amorphous structure [33]. This phenomenon indicated the destruction of the crystalline structure of the chitosan because of the structure reformation during film formation. One small peak at 28° could also be observed in SCSF, which was absent in PaCSF. This peak could be attributed to the remaining minerals [40], because SC was isolated from shrimp shell that contained a lot of minerals (like CaCO_3_).

#### 3.1.3. Surface Morphology

**Figure 3:**
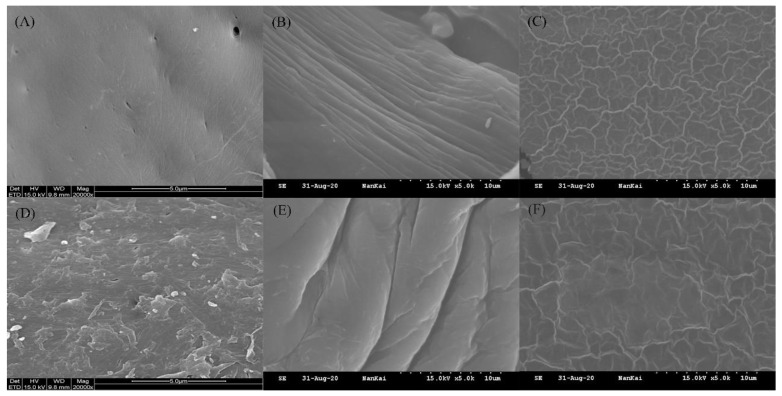
Surface morphology of (**A**) PaC, (**B**) PaCS, (**C**) PaCSF, (**D**) SC, (**E**) SCS, and (**F**) SCSF.

The photographs of the surface morphology of chitin, chitosan, and chitosan films are exhibited in Figure 3. The surface of PaC was smooth with pores and fibrous veins on it; while that of SC was rough. Different species and the isolation methods would contribute to various surface morphology of chitin [41]. PaCS and SCS both had wrinkles on their surfaces, which might result from the same harsh conditions of deacetylation. We could see lots of similar ridge-like grains on the surfaces of PaCSF and SCSF. Since the chitosan films had been kept in a desiccator after being dried, the lack of water might result in these grains.

The results of FTIR, XRD, and SEM showed that chitin, chitosan, and chitosan films from *P. americana* were similar to their counterparts derived from shrimp. However, the Mv and the DDA of PaCS were different from those of SCS, which might influence the performances relating to food packaging of the chitosan films.

### 3.2. Performances of Chitosan Films

#### 3.2.1. Film Thickness and Optical Performance

The film thickness is presented in Table 2. PaCSF was significantly thicker than SCSF (*p* < 0.05). The Mv of PaCS was much lower than that of SCS (*p* < 0.05), as shown in Table 1, meaning shorter polysaccharide chains. The shorter the chains were, the more hydrogen bond sites hiding deeply inside would be exposed, which would then be bound to water molecules. Therefore, during film formation, more water molecules were attracted to PaCS, resulting in the film swelling. Similar result could be observed in Table 2, which showed that water content of PaCSF was significantly higher than that of SCSF (*p* < 0.05). Since the films had lost a lot of water molecules in the desiccator, the initial water content that PaCSF surpassed SCSF would be more considerable.

**Figure 4:**
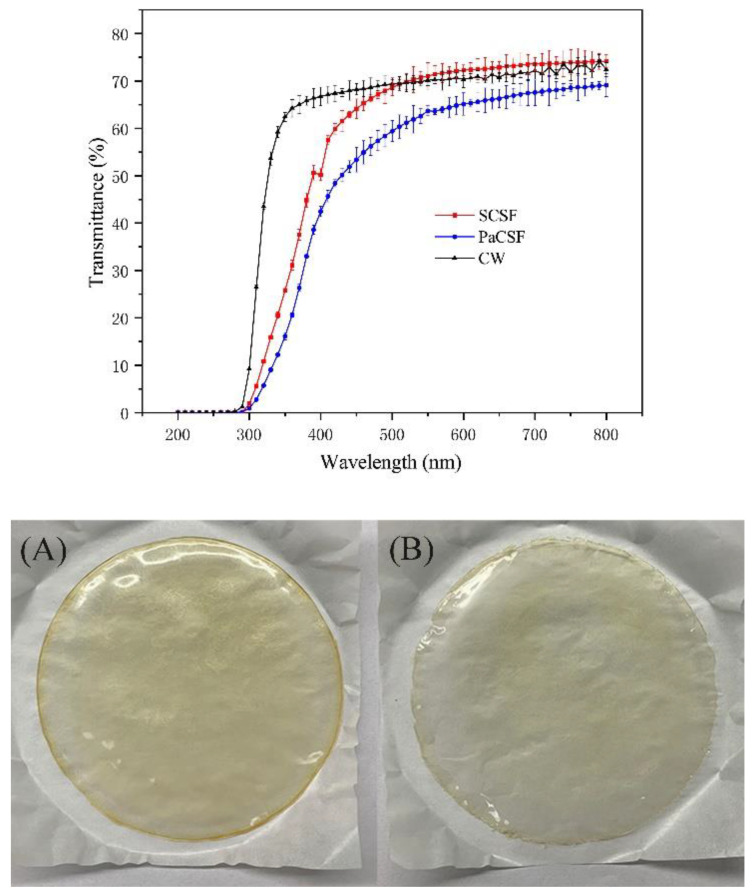
The transmittance of lights from 200 to 800 nm through chitosan films and CW, and the appearances of (**A**) PaCSF, and (**B**) SCSF.

Optical performance was important for films applied in food packaging, especially the ability of UV light resistance [31]. The appearances and the light transmittance of the chitosan films and CW are presented in Figure 4. At the same wavelength, light transmittance of PaCSF was significantly lower than those of SCSF and CW (*p* < 0.05), proving better light resistance and lower transparency of PaCSF. Concerning UV light resistance, both chitosan films showed excellent resistance against UVC light (200 to 300 nm), and PaCSF was significantly better (*p* < 0.05). A lot of pigments deposited in the cuticle of *P. americana*, some of which combined with chitin and survived in PaCSF. Therefore, PaCSF was yellower than SCSF, as shown in Figure 4. These pigments might have absorbed most of the UV light that tried to pass through. Although PaCSF was thicker, its opacity (Table 2) was significantly higher than that of SCSF (*p* < 0.05), proving better ability of light resistance.

#### 3.2.2. WVP

**Figure 5:**
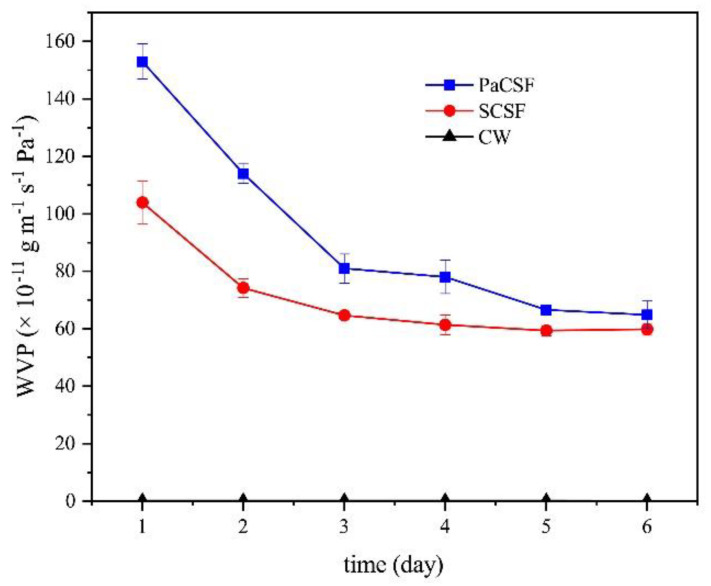
The changes of WVP of PaCSF, SCSF, and CW within 6 days.

As long as the film was intended to be applied in food packaging, its water-proof ability must be assessed. The WVP of the two chitosan films and CW are exhibited in Figure 5. The WVP of the chitosan films were high at the beginning and then dropped, especially in PaCSF case. Since many water molecules had escaped from the chitosan films after drying and storage, a lot of water-binding sites in chitosan films were free to accept water molecules again. As a result, water molecules outside were absorbed by the chitosan films, instead of the calcium chloride anhydrous sealed in the tube. Therefore, the values in the first several days were not exactly the WVP of the chitosan films.

After 6 days, the WVP of PaCSF and SCSF were (64.85 ± 4.82) × 10^−11^ g∙m^−1^∙s^−1^∙Pa^−1^ and (59.81 ± 2.07) × 10^−11^ g∙m^−1^∙s^−1^∙Pa^−1^, respectively. The WVP of PaCSF was higher than that of SCSF (*p* < 0.05), implying relatively weaker water-proof ability of PaCSF. As described in Section 3.2.1, lower Mv of PaCS would result in stronger water-binding ability. Therefore, water molecules were more easily to pass through PaCSF. In previous report, the WVP of the chitosan film was (2.88 ± 0.06) × 10^−11^ g∙m^−1^∙s^−1^∙Pa^−1^ [42], which was significantly lower than those of PaCSF and SCSF (*p* < 0.05). However, in that paper, the molecular weight of the chitosan was 800 kDa, which was much higher than the Mv of PaCS and SCS. Therefore, higher Mv may contribute to better water-proof ability. The WVP of CW was (0.14 ± 0.005) × 10^−11^ g∙m^−1^∙s^−1^∙Pa^−1^, far lower than those of the chitosan films (*p* < 0.05). This result proved that the obtained chitosan films—especially PaCSF—were not good enough in water resistance.

#### 3.2.3. Antioxidant Activity

**Figure 6:**
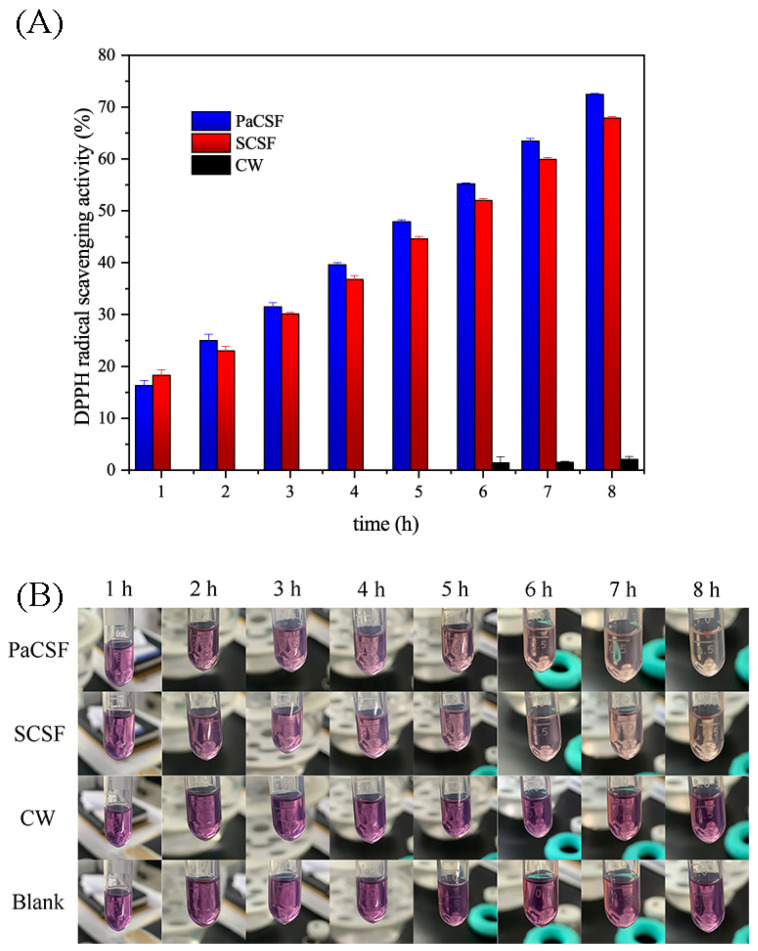
(**A**) DPPH radical scavenging activity and (**B**) the changes of colors of DPPH solutions treated with PaCSF, SCSF, and CW.

DPPH radical scavenging activity was applied to assess the antioxidant activity of the chitosan films and CW. As shown in Figure 6A, DPPH radical scavenging activity of the chitosan films increased linearly during 1–8 h (r^2^ = 0.99, not presented in the figure). At the same time point after 1 h, the antioxidant activity of PaCSF was significantly higher than that of PaCSF (*p* < 0.05). With time passing, the gap between the antioxidant activity of the two chitosan films enlarged. According to Zhang et al. [43], the amino groups of chitosan could form stable macromolecule radicals, contributing to the antioxidant activity of chitosan film. The DDA of PaCS was higher than that of SCS (Table 1), which meant more amino groups in PaCS and higher antioxidant activity. Additionally, in the authors’ opinion, the lower Mv of PaCS also contributed to its higher antioxidant activity. As described in Section 3.2.1, lower Mv of PaCS implied that more amino groups hiding inside the chitosan chains could be exposed to DPPH, which made DPPH radical scavenging easier. DPPH radical scavenging activity of CW was only 2.09 ± 0.53% after 8 h, while that of PaCSF was 72.46 ± 0.16%, indicating significantly better antioxidant activity of PaCSF (*p* < 0.05).

The color changes of DPPH solution treated with different samples are exhibited in Figure 6B, which gave a visual appreciation of the antioxidant activity of the chitosan films. The colors of Blank and CW did not change after 8 h, while those of PaCSF and SCSF bleached out obviously with time passing.

#### 3.2.4. Antibacterial Activity

**Figure 7:**
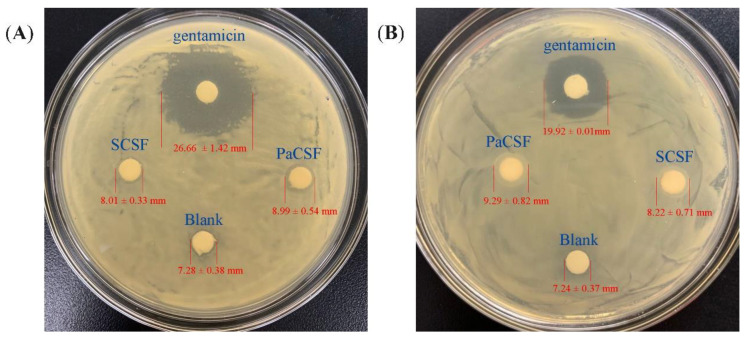
The antibacterial activity of PaCSF, SCSF, Blank, and gentamicin against the growth of (**A**) *Serratia marcescens* and (**B**) *Escherichia coli*.

*Serratia marcescens* and *Escherichia coli* are common foodborne pathogenic bacteria. They were applied to examine the antibacterial activity of the chitosan films. The results are shown in Figure 7. In the case of *S. marcescens*, the inhibition zones of PaCSF, SCSF, Blank, and gentamicin were 8.99 ± 0.54, 8.01 ± 0.33, 7.28 ± 0.38, and 26.66 ± 1.42 mm, respectively. The inhibition zone of PaCSF was significantly bigger than those of SCSF and Blank (*p* < 0.05). Similarly, in the case of *E. coli*, the inhibition zone of PaCSF (9.29 ± 0.82 mm) was also significantly bigger (*p* < 0.05) than those of SCSF (8.22 ± 0.71 mm) and Blank (7.24 ± 0.37 mm). These results indicated that PaCSF possessed better antibacterial activity against *S. marcescens* and *E. coli* than SCSF did.

The antibacterial activity of chitosan derived from its positive charge of protonated amino groups, which would react with the negative charge on the surface of the cells of bacteria, leading to the death of the cells [44]. In addition, as described in Section 3.2.3, the lower the Mv was, the more amino groups would be exposed. Therefore, higher DDA and lower Mv of PaCS (Table 1) enabled its better antibacterial activity against *S. marcescens* and *E. coli* than SCSF did.

## 4. Conclusions

*Periplaneta americana* chitosan film (PaCSF) was produced and characterized. As the results of FTIR, XRD, and SEM showed, PaCSF was similar to shrimp chitosan film (SCSF). However, their performances relating to food packaging were different. PaCSF was thicker because it contained more water. PaCSF could resist UV light more effectively than SCSF did. DPPH radical scavenging assay showed that the antioxidant activity of PaCSF was better than that of SCSF. Antibacterial activity assay exhibited that PaCSF resisted the growth of *S. marcescens* and *E. coli* more effectively than SCSF did. These results implied that *P. americana* was capable of producing chitosan that might be utilized as a raw material for food packaging film without other active additives.

## Figures and Tables

**Table 1:** Different properties of PaC, SC, PaCS, and SCS.

Chitin/Chitosan	DA (%)	DDA (%)	Mv (kDa)	CrI (%)
PaC	74.53 ± 3.28	/	259 ± 10.33	77.33 ± 2.19
SC	71.30 ± 4.46	/	423 ± 18.25	79.72 ± 1.98
PaCS	/	90.85 ± 3.37	16 ± 0.746	40.88 ± 0.65
SCS	/	86.07 ± 2.01	66 ± 4.25	38.62 ± 0.70

Note: “/” in the table means that the value was not determined.

**Table 2:** The performances of PaCSF and SCSF.

Chitosan Film	Water Content (%)	Thickness (mm)	Opacity (A mm^−1^)
PaCSF	42.82 ± 2.31	0.08 ± 0.004	1.19 ± 0.07
SCSF	37.15 ± 3.46	0.06 ± 0.01	0.74 ± 0.06

## Data Availability

No new data were created or analyzed in this study. Data sharing is not applicable to this article.
